# Time course of biochemical, physiological, and molecular responses to field-mimicked conditions of drought, salinity, and recovery in two maize lines

**DOI:** 10.3389/fpls.2015.00314

**Published:** 2015-05-12

**Authors:** Francesco Morari, Franco Meggio, Alice Lunardon, Elia Scudiero, Cristian Forestan, Silvia Farinati, Serena Varotto

**Affiliations:** Department of Agronomy, Animals, Food, Natural Resources and Environment, University of Padova Agripolis Viale dell'UniversitàPadova, Italy

**Keywords:** abiotic stress, drought, maize, salinity, stress response, stress marker genes, stress tolerance

## Abstract

Drought and salinity stresses will have a high impact on future crop productivity, due to climate change and the increased competition for land, water, and energy. The response to drought (WS), salinity (SS), and the combined stresses (WS+SS) was monitored in two maize lines: the inbred B73 and an F1 commercial stress-tolerant hybrid. A protocol mimicking field progressive stress conditions was developed and its effect on plant growth analyzed at different time points. The results indicated that the stresses limited growth in the hybrid and arrested it in the inbred line. In SS, the two genotypes had different ion accumulation and translocation capacity, particularly for Na^+^ and Cl^−^. Moreover, the hybrid perceived the stress, reduced all the analyzed physiological parameters, and kept them reduced until the recovery. B73 decreased all physiological parameters more gradually, being affected mainly by SS. Both lines recovered better from WS than the other stresses. Molecular analysis revealed a diverse modulation of some stress markers in the two genotypes, reflecting their different response to stresses. Combining biochemical and physiological data with expression analyses yielded insight into the mechanisms regulating the different stress tolerance of the two lines.

## Introduction

Drought and salinity are abiotic stresses that reduce plant growth and have a strong impact on crop yield, because they negatively affect both photosynthesis and plant reproduction. In the future these stresses will have a high impact on crop productivity, due to climate change and the increased competition for land, water, and energy (FAO, 2002; Ahuja et al., 2010). In particular, competition for water resources is growing among different social and economic sectors, with agriculture being progressively forced to use lower quality water (Laraus, 2004). This has led to the problem of salinity becoming increasingly serious, particularly near coastal areas where exploitation of the groundwater involves an increased saline intrusion with implications on salt accumulation and soil degradation. Indeed, irrigation-induced salinity represents a main constraint limiting productivity for many crops. Selecting more drought and salt-tolerant genotypes is therefore a desirable way of improving crops (Tester and Langridge, 2010). Maize, one of the most important food, feed and industrial crops, has a pronounced susceptibility to drought and salinity (Bänziger and Araus, 2007): improving the drought resistance of this crop is thus of strategic significance.

A fair amount of studies have focused on comparison of the differential responses of crops to water and salinity stress (e.g., Munns, 2002; Hu et al., 2007; Elmetwalli et al., 2012) as they both lower soil water potential, normally leading to similar physiological responses. The physiological effects of water deficiency on plants are well known: reduction in the photochemical activity of the photosystems (Souza et al., 2004), reduced root adsorption of nutrients from the soil, and slacker roots to shoots nutrient transport (Kramer and Boyer, 1995). Even at high moisture content, soil salinity induces disequilibrium in the ionic ratios in plants (Grattan and Grieve, 1998), resulting in physiological drought with the abovementioned effects (Corwin, 2005). It can also cause specific ion toxicity (Rhoades et al., 1999), and compromise the repartition of macro- and micronutrients within leaves (Neves-Piestun and Bernstein, 2005; Hu et al., 2007).

In many plants, genetic studies have shown that the mechanism underlying drought and salinity stress tolerance is complex. However, its understanding can be facilitated by the adoption of expression analysis approaches to elucidate the molecular basis of stress adaptation and identify the numerous pathways important to growth under limiting water or in saline soil (Bartels and Sunkar, 2005; Shinozaki and Yamaguchi-Shinozaki, 2007; Deinlein et al., 2014). Interestingly, these pathways tend to be conserved among plant species and in fact, one of the most obvious features of adaptation to drought and salt are changes in transcripts profiles for genes involved in many biochemical, cellular, and physiological processes, from transcription regulation to signal transduction, protein biosynthesis and decay, membrane trafficking and photosynthesis (Cabello et al., 2014). From genetic studies it is evident that plant adaptation to drought is a complex biological process that includes up- or down-regulation of specific genes, transient increase in ABA levels, build-up of compatible solutes and protective enzymes, increasing levels of antioxidants and inhibition of energy-consuming pathways (Salekdeh et al., 2009). However, the conservation of pathways and genes is not sufficient to translate results from one species or even genotype to another, because the high conservation of the core gene machinery between plants may not correlate with the expression timing of the stress-induced genes. A diverse stress tolerance between two genotypes may reflect differences in the timing of specific genes up- and/or down-regulation (Skirycz et al., 2011).

Another important aspect of abiotic stress studies in plants is the need to apply stress conditions that retain their value under field conditions, thus improving the translation of research from model plants to crops, for agronomic purposes. In many experiments dealing with stress response, tolerance is assessed predominantly in severe conditions in which plant survival is compromised by a prolonged period of treatment. However, limited resource availability in the field rarely causes plant death, and more favorable environmental conditions usually return after a period of stress, resulting in reduced crop yields (Skirycz et al., 2011; Deikman et al., 2012).

In this study, we analyzed the response to drought, salinity and the combined stresses in two maize genotypes: the reference inbred line B73 for which genomic tools are available and an F1 hybrid selected for its stress tolerance. We developed a protocol to mimic progressive field stress conditions and evaluated the response of the two genotypes during the stress application and after 4 days of recovery. The strategies adopted by the two genotypes to cope with stresses were evaluated using biochemical, physiological, and molecular parameters.

## Materials and methods

### Experimental set-up

The experiment was conducted in May-July 2012 at the experimental farm of the University of Padova, Italy (45°21′ N, 11°58′ E, 6 m a.s.l.). The response to drought and salinity was tested in two varieties of maize (*Zea mays* L.), the hybrid PR32P26 (hereinafter simply called P26, Pioneer Hi-Bred Italia, Gadesco Pieve Delmona, Italy) and the inbred line B73. In a field equipped with an automatic mobile roof to avoid rainfall input, pots (diameter 23 cm, height 23 cm, volume 9500 cm^3^) were filled with a 50%-weight mixture of native sandy loam and silica sand. The resulting substrate (66% sand, 27.5% silt, and 6.5% clay) was sub-alkaline (pH 7.8), had an organic carbon content of 0.40%, and was non-saline (saturated paste electrical conductivity, *EC_e_* = 0.8 dS m^−1^). The substrate was packed in the pots in order to obtain a bulk density of 1.42 ± 3.6 10^−3^ g cm^−3^. Pot water capacity and wilting point were 0.154 ± 1.94 10^−3^ cm^3^ cm^−3^ and 0.072 ± 0.9 10^−4^ cm^3^ cm^−3^, respectively. Before sowing, 0.50 g N, 0.22 g P_2_O_5_, and 0.15 g K_2_O were added to each pot. Maize seeds were pre-germinated for 2 days in wet, rolled paper towels at 25°C, after which three germinating seeds were transferred to each pot. The seedlings were thinned to one per pot after 7 days.

The two varieties of maize were tested under factorial combinations of two water regimes and two salt concentrations, in four treatments: C (non stressed plants, the control), WS (water stress caused by water deficit), SS (salinity stress), and WS+SS (water and salinity stress combined). The experimental design was a randomized block with 3 replications. Since destructive plant samplings were performed on 5 dates, a total of 120 pots were prepared (3 replicates × 4 treatments × 5 times × 2 varieties).

The pots were weighed daily during the experiment. Water non stressed plants (C) were grown at a water content of 100% available water capacity, replenishing the water lost by evapotranspiration every day. On the contrary, water stressed plants (WS) were watered replenishing only 60% of daily evapotranspiration to a minimum water content threshold of 0.10 cm^3^ cm^−3^ (i.e., 40% of the available water capacity). The saline water (electrical conductivity = 20 dS m^−1^) consisted in a controlled mix of ions (Cristal Sea Marinemix®: 54.92% Cl^−^; 30.82% Na^+^; 7.68% SO^2−^_4_; 3.81% Mg^2+^; 1.21% Ca^2+^; 1.12% K^+^; 0.44% NaHCO_4_) reproducing saline groundwater typically found in the coastal soils to the south of the Venice Lagoon, Italy (Scudiero et al., 2012). WS+SS plants were watered replenishing only 60% of daily evapotranspiration as for WS treatment, but with saline water. The use of an equivalent ion concentration in saline water implied that the quantity of ions was lower in the pots of WS+SS treatments compared to SS.

The saline and drought stresses were started June 18th on plants at V6 stage. Until that day, water content in all pots was maintained at pot water capacity.

Plants were sampled at the beginning of the treatments (T0), on June 20th (T2), June 22nd (T4), June 28th (T10) at the end of treatment, and July 2nd (T14) after 4 days of recovery. To verify the plant recovery capacity from water and salinity stress conditions, from June 28th to July 2nd all treatments were watered twice daily with non-saline water, up to a water content of 0.30 cm^3^ cm^−3^ in order to promote salt leaching and optimal soil moisture status.

### Physiological analyses

Single-leaf gas exchange measurements were performed with a LI-6400 portable photosynthesis system (Li-Cor Inc. Lincoln, Nebraska, USA). Analyses were conducted using a circular 2 cm^2^ leaf cuvette equipped with a 6400-40 fluorometer as light source. Measurements were subjected to at least 10-min acclimation at a constant saturating photosynthetic photon flux density (PPFD) of 1500 μmol of photons m^−2^ s^−1^, a CO_2_ concentration of 390 μmol mol^−1^ and relative humidity (RH) between 60 and 70% allowing ~1.7 kPa of vapor pressure deficit (VPD) inside the chamber. Block temperature was maintained at 27°C allowing leaf temperature to range between 29 and 36°C. In addition to net assimilation rate (*A*_n_, μmol CO_2_ m^−2^ s^−1^) and stomatal conductance (*g*_s_, mmol H_2_O m^−2^ s^−1^), the incorporated fluorometer allowed determination of the actual photochemical efficiency of photosystem II (φPSII). This was determined by measuring steady-state fluorescence (*F_s_*) and maximum fluorescence during a light-saturating pulse of *c*. 8000 μmol m^−2^ s^−1^ (*F*′_*m*_) following the procedures of Genty et al. (1989): φPSII = (*F*′_*m*_ − *F_s_*)/*F*′_*m*_.

Measurements were performed on at least three fully expanded leaves per treatment at regular time points during the experiment, between 11.00 a.m. and 2.00 p.m. solar time.

### Chemical analyses on plants and soil

Once physiological analyses had been performed, plants were weighed and analyzed for ions composition and soil was sampled for salinity assessment.

Roots and shoots were dried at 60°C for 48 h and dry matter was measured. Powered biomass was analyzed for cation (Na^+^, K^+^, Mg^2+^, Ca^2+^, and NH^+^_4_) and anion (Cl^−^, SO^2−^_4_, and PO^3−^_4_) by ion chromatography (ICS 900, Dionex, Sunnyvale, CA, USA) according to Nicoletto et al. (2013).

The soil in the pots was air dried and sieved at 0.5 cm and then analyzed for saturated paste electrical conductivity (*EC_e_*) (Rhoades et al., 1999). The osmotic potential of the saturated extract was then analyzed with the WP4-T Dewpoint PotentiaMeter (Decagon Devises Inc., Pullman, WA, USA).

### Real time quantitative PCR (qRT-PCR) analysis

The last expanded leaf was collected between 11.00 a.m. and 12.00 p.m. solar time for RNA extraction. Three biological replicates were used for the two time points (on June 28th T10 and July 2nd T14) of each treatment: C, WS, SS, and WS+SS. Biological replicates were pooled together and total RNA was extracted from maize leaves using the RNeasy Plant Mini Kit (QiAgen) and subjected to on-column DNase treatment (QiAgen). cDNA synthesis was performed with the SuperScript III reverse transcriptase kit (Invitrogen), according to the manufacturer's instructions. One microgram of total RNA was used as a template together with 1 μl oligo (dT)_12−18_ (0.5 μg/μl – Invitrogen). Quantitative Real-Time PCR expression analysis was performed using a StepOnePlus™ Real-Time PCR System (AppliedBiosystems) and the FAST SYBR® GREEN PCR MasterMix (Life Technologies), following the manufacturer's guidelines. Real-time conditions were: 20 s at 95°C, 40 cycles of: 3 s at 95°C and 30 s at 60°C. For each reaction, we observed product melting curves by heating from 60 to 95°C at 0.2°C/s. For all transcripts, this procedure allowed identification of a single product, which we confirmed by analysis on 2% agarose gel. Three technical replicates were done for each primer combination. The constitutively expressed *GAPC2* gene was used as housekeeping internal control of the cDNA/RNA quantity (Russell and Sachs, 1989). Relative quantification of gene expression (normalized to *GAPC2* transcript quantities) was performed with the Pfaffl method (Pfaffl, 2001) using previously determined amplification efficiencies for each gene. Specific primers were designed using Primer BLAST (http://www.ncbi.nlm.nih.gov/tools/primer-blast/) or were selected from published papers. Primer sequences are reported in Table S1 in Supplementary Material.

### Statistical analyses

A Three-Way ANOVA (mixed model with repeated measures) by maize variety, salinity level, and water regime was used to analyze agronomic and physiological parameters. Comparison between means was performed by adjusted Tukey's test.

In order to estimate a possible linear relationship between parameters the Pearson correlation coefficient was calculated. The general structure of the interdependences existing between physiological response, plant growth, chemical composition, and gene expression was finally evaluated performing a correlation-based principal component analysis (PCA) on 12 variables measured before (T10) and after the recovery (T14): leaf dry matter, leaf and root Na^+^, leaf Cl^−^, ratio K^+^/Na^+^ in root, net assimilation (*A*_n_), expression patterns for *PMP3-4, HSP70, CAT1, CoAred*, and *SUS*. Variables were selected according to Kaiser's measure of sampling adequacy (MSA). The overall MSA was 0.74 indicating that PCA was suitable (Kaiser, 1974). Rotated orthogonal components (varimax normalized method of rotation) with eigenvalues >1 were extracted (Kaiser, 1960) and the relative scores were determined.

Statistical analyses were performed with STATISTICA 7.0 (Statsoft Inc., Tulsa, OK, USA) and SAS 9.3 (Cary, NC, USA).

## Results

### Plant development in response to stress

**Figure 1:**
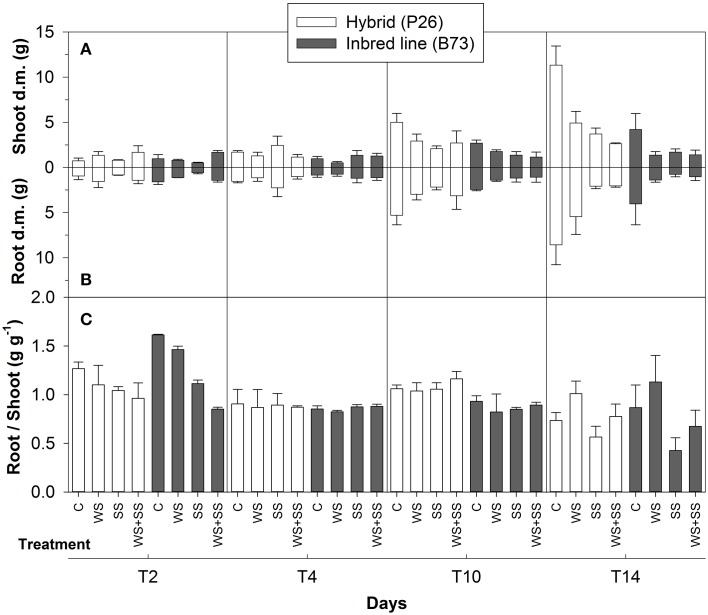
**Shoot (A) and root (B) dry matter obtained from maize plantlets of the hybrid P26 and inbred line B73 grown for 2, 4, 10 days under control (C), drought (WS), salinity (SS), or the combination of drought and salinity (WS+SS) and after 4 days of recovery from the stresses**. In **(C)** Root-to-Shoot ratio (R/S) in the two genotypes and treatments are reported within each time points. Values represent means ± SE (*n* = 3).

To analyze the effect of the stress on plant growth we measured both shoot and root dry matter of control and stressed plants of the two genotypes, during stress applications (at T2, T4, and T10) and after recovery from the stresses (T14). Considering the biomass accumulation at the different time points, the genotypes differed in their growth capacity, the hybrid being more productive than B73 inbred for both shoots and roots (*P* < 0.01; Table 1 and Figure 1).

**Table 1:** **Agronomic and physiological parameters measured during stress application at T2, T4 and T10 and after 4 days of recovery (T14) in wild-type (B73) and hybrid (P26) plants**.

	**EC_S_ μS cm^−1^**		**Soil ψ_π_ MPa**		**Shoot dm g**		**Root dm g**		**Leaf ψ_*t*_ MPa**		**Leaf ψ_π_ MPa**		***A*_n_ μmol CO_2_ m^−2^s^−1^**		***g*_s_ mmol H_2_O m^−2^s^−1^**		**Φ_PSII_ Efficiency**	
**VARIETY**																		
*Wild*	4585	*ns*	−.31	*ns*	1.48	*b*	1.37	*b*	−2.30	*ns*	−2.41	*ns*	14.06	*b*	0.10	*b*	0.09	*ns*
*Hybrid*	5675	*ns*	−0.35	*ns*	2.90	*a*	2.65	*a*	−2.35	*ns*	−2.06	*ns*	17.55	*a*	0.12	*a*	0.10	*ns*
**WATER SALINITY**																		
*No salt*	1736	*b*	−0.24	*a*	2.66	*a*	2.57	*a*	−2.27	*ns*	−1.91	*a*	21.64	*a*	0.14	*a*	0.12	*a*
*Salt*	8524	*a*	−0.43	*b*	1.73	*b*	1.46	*b*	−2.37	*ns*	−2.55	*b*	9.97	*b*	0.07	*b*	0.06	*b*
**SOIL WATER CONTENT**																		
*60%*	4050	*ns*	−0.30	*ns*	1.79	*b*	1.75	*ns*	−2.29	*ns*	−2.34	*ns*	13.06	*b*	0.09	*b*	0.08	*b*
*100%*	6209	*ns*	−0.36	*ns*	2.60	*a*	2.27	*ns*	−2.35	*ns*	−2.12	*ns*	18.55	*a*	0.13	*a*	0.11	*a*
**DAY**																		
*2*	4133	*b*	−0.28	*b*	1.07	*c*	1.19	*c*	−1.70	*a*	−2.09	*ab*	19.57	*ns*	0.13	*a*	0.11	*ns*
*4*	5942	*b*	−0.31	*b*	1.33	*c*	1.23	*c*	−2.90	*b*	−2.25	*ab*	15.26	*ns*	0.10	*ab*	0.09	*ns*
*10*	9593	*a*	−0.55	*c*	2.46	*b*	2.46	*b*	−2.66	*b*	−2.83	*b*	13.93	*ns*	0.09	*b*	0.09	*ns*
*14*	851	*c*	−0.20	*a*	3.91	*a*	3.17	*a*	−2.03	*a*	−1.75	*a*	14.45	*ns*	0.11	*a*	0.09	*ns*

*Values reported in columns represent for each factor the mean of different treatments. Means within each factor followed by different letters are significantly different (P < 0.05) according to Tukey's test*.

Shoot dry matter accumulation indicated that the P26 hybrid coped better with the stress conditions than the B73 inbred line (Figure 1). In the hybrid, both drought (WS) and salinity (SS) reduced shoot growth compared with control treatment (C), however, it was stopped in WS+SS (Figure 1A). Similarly, the growth of B73 shoots was more affected by WS+SS than WS and SS (Figure 1A).

WS influenced root growth, with a reduction of almost 50% compared with control plants in both the hybrid and inbred line (Figure 1B). During stress applications, root growth in P26 was less reduced in WS and WS+SS, the effects of which were similar if compared to C. In this genotype, SS blocked root growth (Figure 1B). Both SS and WS+SS arrested root growth in B73, whereas it was only reduced in WS (Figure 1B).

The effect of salinity stress was evident in B73 SS and WS+SS and determined a decrease in R/S ratio, after 2 days of treatments. At T10, the difference in R/S ratio between the B73 inbred and P26 hybrid was evident with lower ratio in B73 (Figure 1C).

The two genotypes showed a different capacity to recover from the stresses. The shoots of hybrid plants increased growth soon after WS and SS were removed, whereas the removal of WS+SS did not promote shoot growth (Figure 1A). The shoot growth of B73 plants did not change after stress removal and even decreased in WS (Figure 1A). WS removal affected the root growth of P26 hybrid plants, which accelerated after the recovery (Figure 1B). Conversely, root dry matter of the hybrid decreased after WS+SS removal and did not vary at all in SS recovery (Figure 1B). No increase in root dry matter was observed in B73 plants after recovery from any of the stresses (Figure 1B). After the recovery, a significant decrease in the R/S ratio was observed in both genotypes, due to a higher biomass allocation in the shoots. This decrease was particularly evident after the SS and WS+SS recovery. In B73, water availability in the soil favored an increase of R/S ratio after WS recovery, indicating a higher biomass allocation to the root (Figure 1C).

These results indicated that WS and WS+SS reduced the hybrid shoot and root growth compared to C, whereas SS completely inhibited the growth of this genotype that showed a lower recovery capacity in terms of dry matter at T14. B73 plant shoots and roots did not grow during SS and WS+SS and recovery. Relatively more tolerance to WS was shown by the inbred line, however, it was unable to recover as much as the hybrid at T14.

### Ion contents

**Figure 2:**
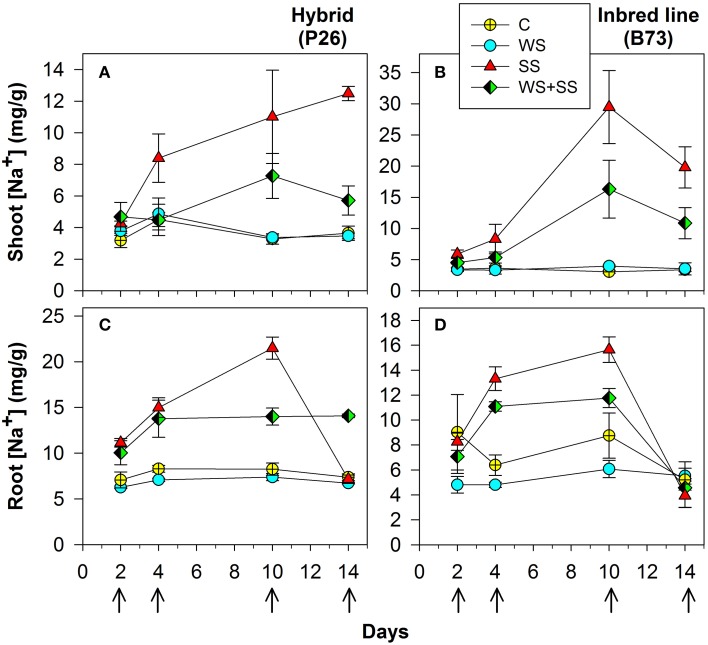
**Shoot (A) and (B), and roots (C) and (D) Na^+^ concentration in maize plantlets of the inbred line B73 and hybrid P26 grown for 10 days under control (C), drought (WS), salinity (SS), or the combination of drought and salinity (WS+SS) and after 4 days of recovery from the stresses**. Values represent means ± SE (*n* = 3). Arrows represent sampling times throughout the experimental period.

**Figure 3:**
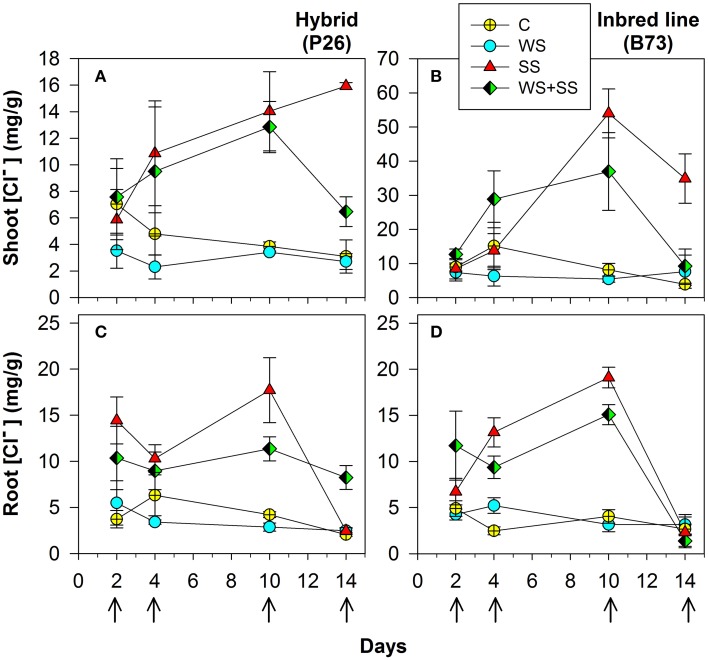
**Shoot (A) and (B) and roots (C) and (D) Cl^−^ concentration in maize plantlets of the inbred line B73 and hybrid P26 grown for 10 days under control (C), drought (WS), salinity (SS), or the combination of drought and salinity (WS+SS) and after 4 days of recovery from the stresses**. Values represent means ± SE (*n* = 3). Arrows represent sampling times throughout the experimental period.

To verify the mechanisms of uptake and translocation of ions in the two genotypes, we measured the ion contents in both shoots and roots during stress application at (T2), (T4) and (T10) and after 4 days of recovery (T14) in B73 and hybrid plants (Figures 2, 3 and Table 2).

**Table 2:** **Shoot and root ion contents measured during stress application at T2, T4, and T10 and after 4 days of recovery (T14) in wild-type (B73) and hybrid (P26) plants**.

	***Shoot (S)* [mg g^−1^]**																***Root (R)* [mg g^−1^]**																**Na^+^**		**Cl^−^**	
	**Na^+^**		**K^+^**		**NH^+^_4_**		**Mg^2+^**		**Ca^2+^**		**Cl^−^**		**PO^3−^_4_**		**SO^2−^_4_**		**Na^+^**		**K^+^**		**NH^+^_4_**		**Mg^2+^**		**Ca^2+^**		**Cl^−^**		**PO^3−^_4_**		**SO^2−^_4_**		***S*/*R***		***S*/*R***	
**VARIETY**																																				
*Wild*	8.02	*a*	12.73	*ns*	1.65	*ns*	4.96	*a*	9.23	*a*	16.36	*a*	1.00	*ns*	0.70	*a*	7.89	*b*	5.01	*ns*	0.88	*ns*	2.53	*ns*	6.93	*ns*	6.80	*ns*	0.61	*ns*	3.27	*ns*	1.29	*a*	5.94	*a*
*Hybrid*	5.53	*b*	13.40	*ns*	1.73	*ns*	3.65	*b*	6.62	*b*	7.11	*b*	0.74	*ns*	0.31	*b*	10.32	*a*	5.12	*ns*	0.85	*ns*	2.32	*ns*	7.54	*ns*	7.15	*ns*	0.49	*ns*	3.54	*ns*	0.57	*b*	1.43	*b*
**WATER SALINITY**																																				
*No salt*	3.62	*b*	12.32	*ns*	1.31	*b*	4.06	*b*	7.59	*b*	5.85	*b*	0.89	*ns*	0.40	*ns*	6.82	*b*	5.75	*a*	0.95	*ns*	2.41	*ns*	7.31	*ns*	3.78	*b*	0.55	*ns*	3.20	*ns*	0.57	*b*	1.75	*b*
*Salt*	9.93	*a*	13.82	*ns*	2.07	*a*	4.55	*a*	8.26	*a*	17.62	*a*	0.84	*ns*	0.61	*ns*	11.40	*a*	4.38	*b*	0.78	*ns*	2.43	*ns*	7.16	*ns*	10.17	*a*	0.55	*ns*	3.61	*ns*	1.29	*a*	5.61	*a*
**SOIL WATER CONTENT**																																				
*60%*	5.56	*b*	11.81	*b*	1.60	*ns*	4.12	*ns*	7.51	*b*	10.17	*b*	0.78	*ns*	0.45	*ns*	8.44	*b*	4.84	*ns*	0.86	*ns*	2.27	*b*	7.20	*ns*	6.66	*ns*	0.48	*ns*	2.97	*b*	0.88	*ns*	2.80	*ns*
*100%*	7.99	*a*	14.33	*a*	1.78	*ns*	4.49	*ns*	8.35	*a*	13.30	*a*	0.96	*ns*	0.57	*ns*	9.77	*a*	5.29	*ns*	0.87	*ns*	2.57	*a*	7.27	*ns*	7.29	*ns*	0.62	*ns*	3.83	*a*	0.98	*ns*	4.57	*ns*
**DAY**																																				
*2*	4.15	*b*	15.55	*a*	1.78	*ns*	4.40	*b*	8.50	*b*	7.69	*b*	1.12	*ab*	0.66	*a*	7.97	*c*	6.53	*a*	1.04	*ns*	2.58	*a*	7.08	*b*	7.70	*ab*	0.80	*a*	4.60	*a*	0.57	*b*	1.44	*b*
*4*	5.36	*b*	15.58	*a*	1.83	*ns*	2.83	*c*	4.03	*c*	11.44	*b*	0.55	*b*	0.27	*b*	9.97	*b*	5.87	*a*	0.96	*ns*	2.70	*a*	8.01	*ab*	7.41	*b*	0.46	*ab*	3.75	*a*	0.57	*b*	1.93	*b*
*10*	9.72	*a*	12.01	*b*	1.60	*ns*	5.69	*a*	10.19	*a*	17.33	*a*	1.22	*ab*	0.77	*a*	11.67	*a*	4.51	*b*	0.72	*ns*	2.75	*a*	8.43	*a*	9.69	*a*	0.60	*ab*	3.60	*a*	0.78	*b*	1.70	*b*
*14*	7.87	*a*	9.12	*b*	1.53	*ns*	4.29	*b*	9.00	*b*	10.49	*b*	0.59	*ab*	0.33	*b*	6.82	*c*	3.36	*b*	0.74	*ns*	1.65	*b*	5.42	*c*	3.10	*c*	0.33	*b*	1.66	*b*	1.80	*a*	9.67	*a*

*a Values reported in columns represent for each factor the mean of different treatments. Means within each factor followed by different letters are significantly different (P < 0.05) according to Tukey's test*.

Na^+^ concentration was significantly higher in both shoots and roots of hybrid and B73 plants grown under SS and WS+SS compared with WS and C treatments (Figure 2 and Table 2).

At T10, Na^+^ concentration in roots of hybrid plants grown under SS and WS+SS treatments was about three and two times higher, respectively, than in plants grown under WS and C (Figure 3C) and it was lower compared to B73. At the same time point, Na^+^ root concentration in B73 was about three and four times higher in SS and WS+SS, respectively than in WS and C (Figure 2D).

Considering the effect of recovery in the hybrid, it is interesting to note that the Na^+^ concentration in roots dropped to the same value as C after SS, while recovery had no effect after WS+SS (Figure 2C). An opposite Na^+^ concentration trend was observed in hybrid shoots (Figure 2A). The recovery had no effect on leaves grown under SS and a decrease in Na^+^ concentration was instead observed in shoots grown under WS+SS. In B73 plant roots grown under SS and WS+SS, Na^+^ concentration dropped to the level in non-treated and WS treated plants after the recovery, while in shoots grown under both SS and WS+SS a reduced concentration of Na^+^ was observed (Figure 2B). However, Na^+^ concentration remained four times (SS) and two times higher (WS+SS) than that measured in C and WS plant shoots. Factor analysis revealed that the ratio between leaf Na^+^ and root Na^+^ differed significantly between the hybrid and B73: 0.57 and 1.29 respectively; similar ratio differences were obtained considering only the salinity effect (Table 2). Interestingly, the ratio increased significantly from 0.78 to 1.80 (*P* < 0.01) after the recovery (Table 2).

In plants grown under C and WS, Cl^−^ concentrations were very similar for the two genotypes, and no significant variations were found over 10 days of stress application in shoots and roots (Figure 3 and Table 2). However, when plants were grown in SS and WS+SS a significant increase in Cl^−^ concentration was found in the shoot. An evident difference in Cl^−^ concentrations was observed between the shoots of the hybrid (14 mg/g) and those of the inbred line (50 mg/g; Figures 3A,B). Conversely, Cl^−^ concentrations in the roots of the two genotypes were quite similar (Figures 3C,D). After 4 days of recovery from WS+SS, the Cl^−^ concentration was reduced by about 50% in the hybrid leaves whereas it continued to increase during the recovery from SS (Figure 3A). In the B73 shoots Cl^−^ concentrations decreased during recovery from both SS and WS+SS, but the ion amount remained higher in SS compared to the other treatments (Figure 3B). The effect of recovery from these stresses also led to a reduction in Cl^−^ concentrations in the root of the two genotypes (Figures 3C,D). As observed for Na^+^, the repartition of Cl^−^ between shoot and root was also significantly different between the hybrid and B73, with a ratio of 1.43 and 5.94, respectively (Table 2). Potassium (K^+^) concentration in shoots and roots was unaffected by treatments with the exception of SS (Table 2). SS increased concentrations of the other cations, NH4^+^, Mg^2+^ and Ca^2+^, in the shoots of the two genotypes while no significant effects were observed for roots (Table 2). Moreover, leaf Mg^2+^ and Ca^2+^ concentrations were both affected by the variety, with higher values in B73 than the hybrid; the opposite was observed for these two cations in the root (Table 2). The effect of recovery was significant for the concentrations of K^+^, Mg^+^, Ca^2+^ in the leaf and Mg^2+^ Ca^2+^ PO^4−^, and SO^4−^ in the root (Table 2).

Taken together these data showed that the two genotypes have different ion accumulation and translocation capacity when subjected to stress conditions. This is particularly evident in the case of Na^+^ and Cl^−^ accumulation in roots and leaves of the two genotypes grown under SS.

### Photosynthetic parameters

**Figure 4:**
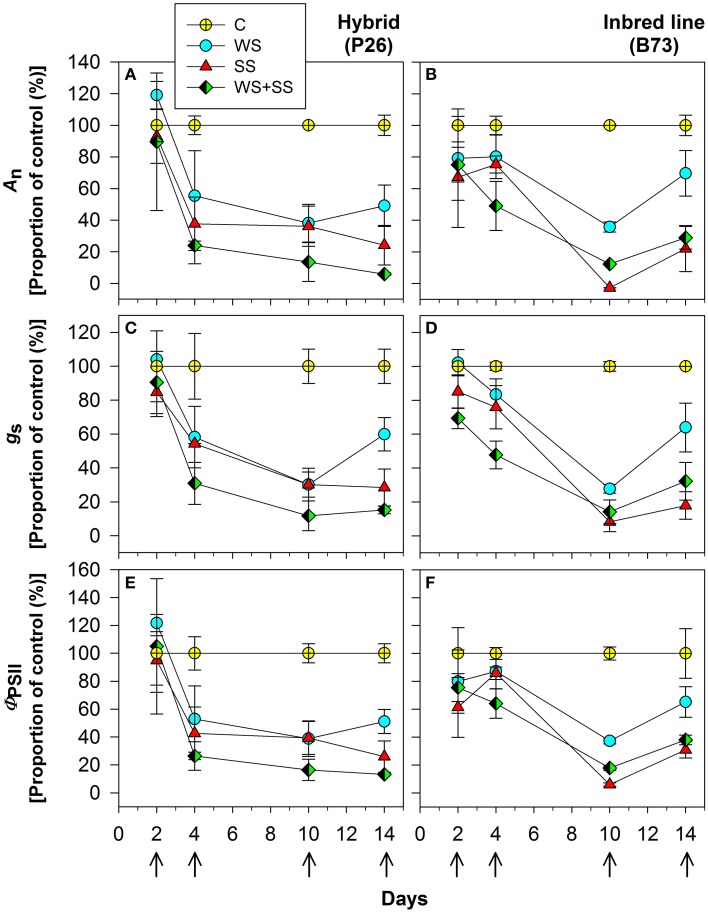
**Effect of water stress (WS), salt stress (SS), and their combination (WS+SS) on the (A,B) net CO_2_ assimilation (A_n_) and (C,D) stomatal conductance (g_s_) and (E,F) PSII quantum efficiency (Φ_PSII_) *for P26 (left) and B73 (right) genotype plants***. Average ± SE values of A_n_, g_s_, and Φ_PSII_ are expressed as a proportion of the control. Arrows represent sampling times throughout the experimental period.

**Figure 5:**
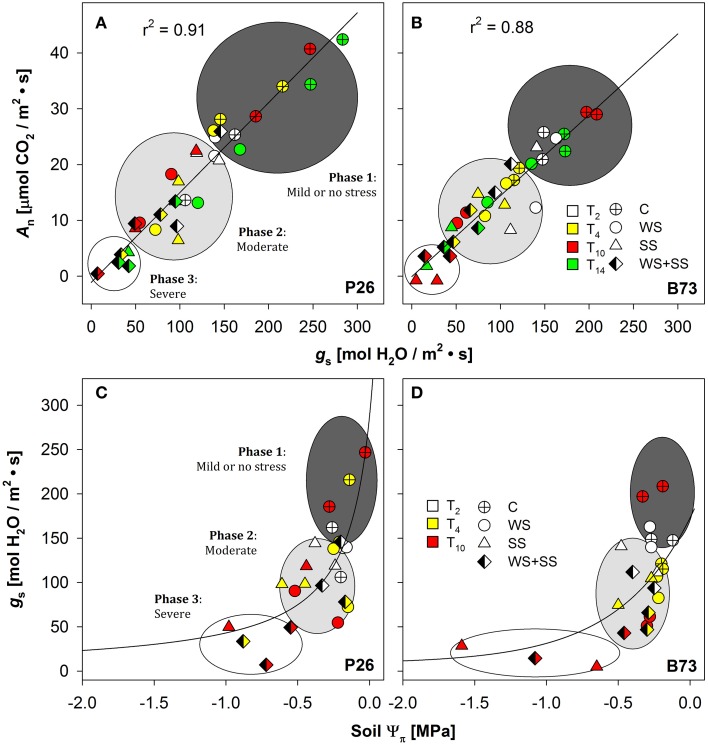
**Stomatal conductance (g_s_) in (A) P26 [*y* = −2.75 + (265.79 · *x*)/(1367.33 + *x*), *R*^2^ = 0.91] and in (B) B73 [*y* = −0.89 + (220.5 · *x*)/(1312.94 + *x*), *R*^2^ = 0.88] as a function of net CO_2_ assimilation rate (A_n_), or soil osmotic potential (Ψπ) in (C) P26 [*y* = 287.8 · −0.18/(−0.18 + *x*), *R*^2^ = 0.54] and in (D) B73 [*y* = 299.73 ·− = 0.16/(-0.16 + *x*), *R*^2^ = 0.37] in well-watered (C), water-stressed (WS) salt-stressed (SS) and their combination (WS+SS) plants of the two genotypes, P26 and B73**. Each color corresponds to measurements at different time points (T0, 2, 4, 10, and 14). The curve of best fit for **(A,B)** and **(C,D)** plots was a single rectangular hyperbola and a hyperbola decay function, respectively. Three main regions are distinguished along the curves using g_s_ as a reference parameter: mild or no stress (Phase 1), moderate (Phase 2) or severe stress (Phase 3).

To determine the physiological response of plants to the stresses, net assimilation, stomatal conductance, and quantum efficiency of photosystem II were studied (Figures 4, 5; Table 1). Net assimilation (*A*_n_) measured in the control condition (C) was 19.48 ± 5.85 and 23.33 ± 2.43 μmol CO_2_/(m^2^ • s) for P26 and B73 genotypes, respectively. At the same time, stomatal conductance (*g*_s_) and quantum efficiency of photosystem II (Φ_PSII_) were 133.99 ± 27.95 mol H_2_O/(m^2^ • s) and 0.10 ± 0.03, respectively for P26 and 148.10 ± 19.68 mol H_2_O/(m^2^ • s) and 0.13 ± 0.02 for B73. As a consequence of WS, SS and their combination WS+SS, *A*_n_, *g*_s_, and Φ_PSII_ decreased in both genotypes, as shown on a percentage of control basis in Figure 4. The stress effect was already evident at early stages (T4) in P26 with reductions of ~60% for all parameters measured compared to C. In B73, on the contrary, while only a small reduction (~20%) was measured at T4 for WS and SS treatments, for WS+SS the effect was stronger leading to a halving of all three parameters. When stress conditions became more severe (T10) their effect was progressively higher in B73 than in P26, becoming evident and statistically significant between genotype and treatment. After 10 days, no significant differences were measured between genotypes for WS and WS+SS treatments. Under SS, while values similar to those for WS were measured in P26, an almost complete inhibition of photosynthetic apparatus (*A*_n_, Φ_PSII_) and quasi-complete stomatal closure (*g*_s_) were detected in B73. At T14, a recovery capacity upon re-watering up to values of 50–70% compared to C was measured for both genotypes under WS. Under SS and WS+SS, while B73 demonstrated a recovery, although small, to values of 30–40%, P26 showed no significant differences from the previous time point (T10) for both treatments, leading to values of about 20–30% compared to C. These results indicated that the response of the two genotypes to the applied stresses is physiologically different: at T4 the hybrid perceived the stress, reduced all the analyzed physiological parameters, particularly in WS+SS, and kept them reduced until the recovery, when it reacted better to WS compared to the other stresses. B73 decreased all physiological parameters more gradually until T10, being mainly affected by SS, and recovered immediately after the stress removal, especially from WS.

The dependence of *A*_n_ on *g*_s_ (i.e., their ratio or leaf intrinsic water use efficiency, iWUE_leaf_) was analyzed, as well as of *g*_s_ on soil osmotic potential (soil **Ψπ**). Data comprising C, WS, SS and WS+SS for both genotypes are presented in Figure 5, and the best-fitting regression curves are shown. When *g*_s_ was plotted against *A*_n_ (Figures 5A,B) and against soil Ψ_0_ (Figures 5C,D) a linear and exponential growth function, respectively, satisfactorily fitted data from both genotypes. The evaluation of these regressions enabled the detection of three distinct phases: “mild or no stress,” “moderate stress,” and “severe stress” (Figure 5). The results revealed a similar pattern of photosynthetic response to both WS and SS and their combination WS+SS, but with different ranges between the two genotypes. In the early stages of the mild or no stress phase, A_n_ values for P26 were higher than those detected for B73 (Figures 5A,B). After an early stress effect resulting in partial stomatal closure (phase 2, see Figures 5A,B, moderate stress), a further reduction of g_s_ was evident as stress gradually became severe (T10, see phase 3) and an almost complete inhibition of A_n_ for P26 under WS+SS (Figure 5A). In contrast, an even higher stomatal closure leading to a complete inhibition of A_*n*_ was measured for B73 under SS conditions (Figure 5B). When plotting *g*_s_ against soil Ψ π (Figures 5C,D) only plants at T2 and T10 were used and the results revealed a similar pattern of soil Ψ π response to both WS, SS, and their combination (WS+SS), following the same *g*_s_ threshold observed for *A*_n_/*g*_s_ relationship. These results underlined how under severe stress (T10) plants of both genotypes under SS and WS+SS experienced lowest soil **Ψπ** with values of up to ~–1 MPa on average.

### Gene expression analyses

**Figure 6:**
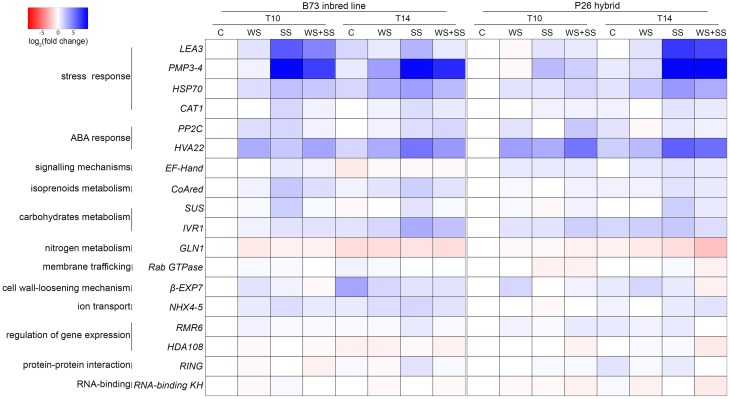
**Heat map representing the relative quantification of gene expression in maize leaves of B73 inbred line and hybrid P26 at two time points, after 10 days of stress (T10) and 4 days of recovery (T14) following the application of drought (WS), salinity (SS), and drought+salinity (WS+SS)**. The maize *GAPC2* gene was selected as internal control. Each experiment was run in triplicate. Data from qRT-PCR experiments were analyzed according to the Pfaffl method and gene expression was calculated as the fold change (FC) relative to the expression level of the control non-stressed sample (C) of the same genotype at T10. Cell colors represent the log_2_(FC) values: blue color for higher relative expression values, red color for lower relative expression values.

To assess whether the diverse stress tolerance of the B73 inbred line and the P26 hybrid is related to a difference in the type and timing of gene up- and/or down-regulation, a gene expression analysis was performed. The transcript level of genes known to be up-regulated by stress or belonging to the main pathways involved in abiotic stress response was analyzed using Real Time Q-PCR. The expression analysis was performed on leaves sampled at two time points: after 10 days of stress application (T10) and after 4 days of recovery (T14) from the stress. For each genotype, gene expression was normalized to the *GAPC2* transcript quantity and then expressed as the fold change relative to the expression level of the control non-stressed sample at T10. For a better understanding of the results obtained, the log_2_ value of these fold changes are shown as colors from red to blue, from negative to positive values (Figure 6). The fold change values are reported in Table S2 in Supplementary Material.

Three stress marker genes, *LEA3, PMP3-4*, and *HSP70*, characterized by a diverse expression pattern in B73 and P26, showed similar trends of expression changes. *LEA3* encodes a member of the late embryogenesis abundant (LEA) proteins, which are major hydrophilic proteins that can reduce the damage caused by adverse environmental conditions (Liu et al., 2013). *PMP3-4* encodes one of the maize plasma membrane proteins 3 (PMP3), which are small molecular weight hydrophobic proteins that respond to abiotic stresses and maintain intracellular ion homeostasis (Mitsuya et al., 2005; Fu et al., 2012). *HSP70* encodes a member of the small heat shock protein family of chaperones, which play an important role in plant stress tolerance (Bartels and Sunkar, 2005). These genes showed the highest induction in response to SS and the combined WS+SS. The increase in expression level was considerably higher for *LEA3* and *PMP3-4* compared to *HSP70*. In SS and WS+SS conditions, in B73 the three genes were up-regulated at T10 during the treatments and their transcript levels decreased at T14 during the recovery. Conversely, in the same conditions, in P26 the up-regulation of these genes at T14 was much higher than that observed at T10. A fourth stress marker gene was analyzed: *CAT1*, encoding a catalase, which eliminates the reactive oxygen species hydrogen peroxide and was previously demonstrated to be drought-induced in maize (Zheng et al., 2010). The two genotypes showed a similar trend of *CAT1* expression: in our conditions the gene was not altered by WS but induced by both SS and WS+SS. During the recovery from the stresses its expression was maintained constant or slightly decreased.

Two genes involved in plant responses to environmental stresses involving ABA were analyzed: the putative *HVA22* gene and the protein phosphatases 2C coding gene *PP2C* (Shen et al., 2001; Bartels and Sunkar, 2005). The putative *HVA22* gene showed similar trends in the two varieties: in both genotypes, *HVA22* was up-regulated in all stress conditions, particularly during WS and WS+SS, and during the recovery its transcript levels increased for SS and decreased for WS and WS+SS. The *PP2C* gene showed some differences in expression trends between the two varieties. WS caused an increase in *PP2C* transcript levels that decreased during the recovery in both genotypes, while SS and WS+SS caused the gene up-regulation only in B73 and P26, respectively. During the recovery, *PP2C* transcript levels did not change for SS in either genotype, while for WS+SS its levels were up-regulated in B73 and down-regulated in P26.

The putative calcium-binding domain *EF-hand* coding gene, the most common protein motif for the binding of Ca^2+^, whose signaling participates in the osmotic and ionic stress responses (Bartels and Sunkar, 2005), was not altered in B73 by the abiotic stresses, while in P26 it was up-regulated by the stresses and its up-regulation was maintained high at T14.

Two genes participating in carbohydrate metabolism were analyzed: *IVR1*, coding for a soluble invertase, known to be up-regulated by drought stress in the maize basal leaf meristem (Kakumanu et al., 2012) and *SUS*, coding for a sucrose synthase, a key enzyme involved in sucrose metabolism and transcriptionally induced during salt stress in maize roots (Wang et al., 2003). In B73, *IVR1* transcript level was up-regulated during SS and WS+SS and did not decrease during the recovery. Instead, in P26 it was up-regulated during all stresses and decreased during the recovery. In B73, *SUS* expression was induced during SS treatment, decreasing during the recovery, while in P26 it was induced by both SS and WS+SS only at T14.

*CoAred* encodes a putative 3-Hydroxy-3-methylglutaryl Coenzyme A Reductase (CoAred), a protein involved in plants isoprenoid metabolism that regulates the synthesis of mevalonic acid (Stermer et al., 1994). In B73 the *CoAred* gene was up-regulated during SS and WS+SS, decreasing its expressing during the recovery, while in P26 it was induced by WS+SS at T10 and by SS only at T14.

Two genes were down-regulated by the applied stresses: the *GLN1* gene encoding a glutamine synthetase, which is repressed in wheat and rice by water stress (Nagy et al., 2013; Singh and Ghosh, 2013) and the *Rab GTPase* that encodes a putative member of the Rab family that plays essential functions in stress signaling (Hong et al., 2013). In B73, *GLN1* was down-regulated during all stresses at T10, while in P26 its expression decreased only at T14, mainly following SS and WS+SS. The *Rab GTPase* expression was slightly decreased only in P26 during SS and WS+SS stresses.

The *ß-EXP7* transcript encodes an expansin isoform. Expansins were suggested to contribute to the fast adjustment of cell wall-loosening in maize under water stress (Geilfus et al., 2010). Both genotypes increased *ß-EXP7* transcription during WS and decreased it during the recovery from all stresses, except from WS in P26.

Tonoplast-associated Na^+^/H^+^ antiporters are responsible for detoxifying the cytoplasm by pumping Na^+^ into the vacuole, improving salt tolerance. In a maize drought-sensitive line they were induced by salt stress in roots (Zorb et al., 2005). In B73, the expression of the antiporters *NHX4* and *NHX5* (here called *NHX4-5* because they were amplified simultaneously by the same primer pair) was induced by all stress treatments at both T10 and T14, while in P26 it was induced only during the recovery from SS and WS+SS.

Finally, the response to stresses was analyzed for two epiregulators, *RMR6* coding for a subunit of Pol IV (Erhard et al., 2009) and *HDA108* coding for a histone deacetylases (Forestan et al., unpublished), for a putative *RING Zn-finger* coding gene and for the putative *RNA-binding KH* domain-containing protein coding gene. With a few exceptions, these genes were not differentially expressed.

Taken together our results indicated that gene expression in the two genotypes was modulated in response to the applied stresses. However, gene expression patterns were not coincident in the two genotypes and reflected, at least in part, their different response to WS, SS, WS+SS and their recovery.

### Principal component analysis (PCA)

The PCA was done to establish the general structure of the interdependences existing between the changes in the levels of genetic stress markers and the fluctuations in the selected environmental parameters associated with WS, SS, and WS+SS (Tables 1, 2). The PCA referred to those markers related to ion homeostasis and the maintenance of cellular osmotic balance: water content in plants (estimated from shoot dry matter), inorganic ions related to stress applications (leaf and root Cl^−^, leaf and root Na+, root K+/Na+ ratios) and An. We also included in the analysis a set of genes markers of stress and belonging to different stress responsive pathways. *PMP3-4, CoAred*, and *SUS* presented dissimilar expression patterns in the two genotypes in response to stress as determined by Q-PCR on the same plant samples; *HSP70* had up-regulation levels mainly related to the type of stress applied; *CAT1* had the same expression pattern in both genotypes and was up-regulated in SS.

Application of PCA to data allowed 3 components to be extracted explaining more than 80% of the total variability. The first component, which accounted for 56% of the variance, was highly correlated (factor loadings ≥> 0.78) with Na^+^ and Cl^−^ contents in leaves and up-regulated stress-responsive genes (*CAT1* and *CoAred*). The second and third components explained 19% and 8.8% of the variance, respectively, and were correlated with Na^+^ and Cl^−^ contents in root (PC2) *PMP3-4* and *HSP70* (PC3).

**Figure 7:**
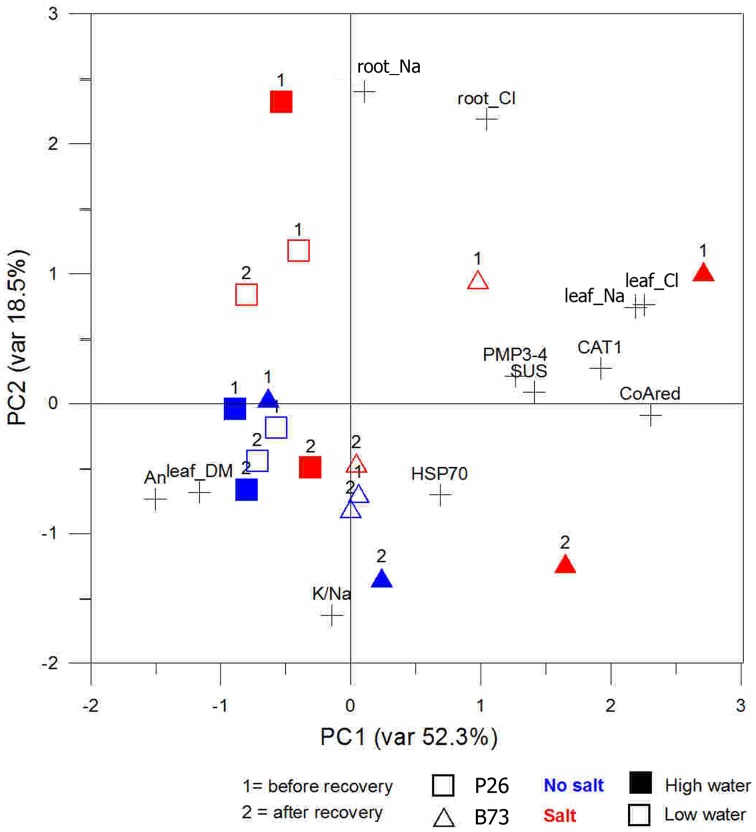
**Site score plot of the studied variables on the two principal components (PC1, PC2)**. PCAs included, as the analyzed variables, those related to osmotic adjustment or those related to gene expression. Plotted points belong to the genotypes (squares and triangles) time points during stress application (1 and 2) and colors to the stress type (blue and red) variables.

Plotting data according to PC1 and PC2 (Figure 7) identified a cluster in quadrant III, including mainly the plants not subjected to SS irrespective of the recovery. They are associated to high *An* and leaf dry matter values. The opposite quadrant (I) groups B73 plants under SS and WS+SS treatments before the recovery. Salt concentration in leaf (Na^+^ and Cl^−^) and expression of *PMP3-4, CAT1*, and *SUS* transcripts are the primary clustering factors. After the recovery, WS+SS B73 is shifted toward the group of non stressed plants in quadrant III while B73 is positioned in quadrant II driven by the reduction of salt concentration in root (PC2 < 0) and persisting high Na^+^ and Cl^−^ concentrations in leaves (PC1 > 1.5). Finally, the hybrid under SS and WS+SS is clustered in quadrant IV by both higher and lower concentrations of Na^+^ and Cl^−^ in root and leaf, respectively. The effect of recovery is depicted by the shift of the hybrid under SS treatment into quadrant III, whereas the hybrid under WS+SS treatment remains unaffected.

The analysis confirmed that the inbred line B73 is very sensitive to SS, indeed more so than the combined WS+SS in our condition. The recovery from WS+SS showed a positive effect on this genotype, while the effect of recovery after SS application was less evident. The analysis also indicated that the hybrid recovered very well from SS and was only slightly affected by WS and WS+SS.

## Discussion

This study was conceived to compare the response of two maize genotypes (the B73 inbred line for which genomic tools are largely available and the P26 commercial hybrid) to a progressive time-limited (10 days) application of drought, salt and a combination of both. These genotypes were already known to have different ability to cope with stress, although the genetic basis of P26 tolerance to stress was not known. Drought and salinity are major abiotic stresses that limit growth and affect crop productivity in many areas of the world. They are caused by the reduced availability of water, increasing use of poor quality water for irrigation and soil salinization (Rozema and Flowers, 2008; Trenberth et al., 2014). This study compared a realistic stress protocol (for salinity alone SS, drought alone WS and combined salinity plus drought WS+SS) simulating a field environment, in which combined salinity plus drought was achieved by watering with a reduced quantity of salted water. At farm level this combination is observed when maize is irrigated with low quality water (e.g., high salt content). As outlined in previous studies, applying realistic protocols, standardizing the measurement and description of plant stresses makes findings more valuable for data comparisons or for translating the findings to crop breeding (Zhang et al., 2004; Nelson et al., 2007; Talame et al., 2007; Skirycz et al., 2011).

To achieve our primary objective we monitored the stress response using a combination of biochemical, physiological and molecular parameters and elaborated the retrieved data sets to depict a complete picture of stress response and recovery capacity of the two genotypes. Firstly, the stress conditions were analyzed in terms of plant growth, indicating that all the applied stresses were effective in limiting both shoot and root growth in the hybrid and arresting the growth in the inbred line. After 4 days from the removal of the stress conditions, results indicated that a longer recovery time is needed for the inbred line shoots to start growing again. Even more complex was the recovery capacity at root level, since no effect on growth after stress removal was observed in either genotype, with the exception of the hybrid following WS. These observations on growth inhibition are consistent with the physiological data on net assimilation, stomatal conductance and quantum efficiency of photosystem II. Furthermore, these data indicated that the tolerance to stress is not necessarily associated to a prompt recovery capacity of a genotype (Nayyar and Gupta, 2006; Efeoğlu et al., 2009). However, it would be important to breed for maize varieties with a high recovery capacity, especially in those regions where drought and salt stress can be of limited duration in the growing season with water availability being restored naturally after a period of drought (Nelson et al., 2007).

Interestingly, we observed that B73 and the hybrid accumulated a similar concentration of Na^+^ at root level; however the concentration was significantly different in the leaves of the two genotypes, suggesting that B73 accumulated a higher level of Na^+^ in the leaf through translocation from the roots during SS and WS+SS. As expected, after the recovery from SS and WS+SS, the Na^+^ concentration in B73 root dropped to C level and clearly decreased in the leaf, although remaining at high levels compared with both C and the hybrid. A very similar trend was observed for the Cl^−^ accumulation in the roots and leaves of the two genotypes. The data on ions uptake and translocation clearly indicated that the different ability to cope with stress, particularly SS and WS+SS, of the two genotypes is somehow associated to different Na^+^ and Cl^−^ translocation dynamics in the shoot. The control of Na^+^ transport by secreting and sequestering it in cellular compartments such as tissues, cells or organelles where Na^+^ is less toxic, is critical to cope better with salinity (Munns and James, 2003; Parida and Das, 2005). Indeed, salinity stress is due to the accumulation of high concentrations of Na^+^ in the leaf cell cytoplasm (Jha et al., 2010). However, Cl^−^ is the main stressful ion in some species (Prior et al., 2007) because they are better at excluding Na^+^ than Cl^−^ (Munns and Tester, 2008). When both Na^+^ and Cl^−^ are taken up in large amounts by the root, they negatively affect plant growth by impairing metabolic processes and decreasing photosynthetic efficiency (Deinlein et al., 2014). Interestingly, in our study a clear relationship exists between Na^+^ and Cl^−^ exclusion and salinity tolerance in P26 hybrid. Further investigations are needed for understanding the mechanisms involved in the uptake and movement of Na^+^ and Cl^−^ throughout the plant of P26 hybrid.

To assess the water and salt stresses actually endured by plants net assimilation, stomatal conductance and quantum efficiency of photosystem II were recorded. These parameters provided precise information on the drought and salt stress intensity occurring in the plant, and allowed three phases to be defined (mild or no stress, moderate and severe) during the progressive application of WS, SS, and WS+SS. The physiological parameters confirmed that P26 was less tolerant to WS+SS and B73 very sensitive to SS, and enabled a more accurate correlation to be established between gene expression variation and stress progression. It has been observed that the kinetics of stress treatments are particularly important and should be carefully considered in experimental designs, especially when expression analyses are performed to identify stress responsive genes (Deyholos, 2010). In our study, the molecular analysis was performed determining the transcript levels of genes that in many previous studies were monitored on samples collected from plants subjected to high-intensity stress treatments, and often a very short time after application of the stress (Kawasaki et al., 2001; Seki et al., 2001; Kreps et al., 2002; Oztur et al., 2002; Rabbani et al., 2003; Atienza et al., 2004; Lan et al., 2005; Rensink and Buell, 2005), whereas we monitored the transcript level at the end of a progressive stress application (T10) corresponding to the severe phase of stress and after 4 days of recovery from the stresses (T14). Therefore, due to our experimental design, gene expression was specifically affected both by the stress duration and severity and it cannot be excluded that some drought and/or tolerance-related genes activated earlier, to prepare the plant for a developing water and salinity stress, were not highly expressed at the considered time points. The transcript level variations observed at these two time points were broad and depended upon both the applied stress and the genotype. In our conditions, some genes were confirmed to be good markers of stress, such as *HVA22* that was up-regulated at T10 and T14 in WS, SS and WS+SS in both genotypes, confirming previous observations in other plant species (Brands and Ho, 2002). *EF-hand* was a good marker of the three stresses in P26 at both time points. *LEA3, PMP3-4*, and *HSP70* represented good markers of SS and WS+SS, but with a distinction between the two genotypes, indicating that they might differently regulate the expression of these genes, commonly expressed in diverse stress conditions (Wang et al., 2004; Liu et al., 2013), as a consequence of their different tolerance to the stress. Some genes appeared to be good markers of salinity stress at least in the more susceptible B73 inbred: *CAT1, CoA-red, SUS*, and *IVR1* transcripts were all up-regulated in SS and less in WS+SS at both T10 and T14, suggesting that 4 days of recovery is not a sufficient time to regain the transcript levels observed in the control. In P26 the transcripts of these genes had more variable trends, highlighting the different response of the two genotypes to the stress at transcriptional level. Previous studies reported that different transcript levels of *CAT1* were detected in stress-susceptible and tolerant maize inbred line (Zheng et al., 2010), that an up-regulation of maize *SUS* was observed a few hours after salt stress application (Wang et al., 2003), and that *IVR1* showed increased transcript abundance in the leaf meristem following drought stress (Kakumanu et al., 2012).

PCA, which was used to combine some selected and correlated parameters, clearly showed the different stress tolerance in the two genotypes: it associated the tolerance of the hybrid to leaf dry matter and *An*. It also correlated the low tolerance of B73 to the Cl^−^ and Na^+^ concentration in leaf and root and to the expression of genes that are good markers of stress for the inbred line. Interestingly, it highlighted the effect of recovery that was evident for the hybrid under SS, whereas there was none under WS+SS.

The ultimate aim of this study was to set up reproducible WS, SS, and WS+SS protocols in which these three time-limited stress conditions could be verified at biochemical, physiological and molecular level, and once set up, to reproduce these stress protocols in further experiments and analyze their effect at epigenetic and genetic genome-wide level. It would be interesting to better dissect the characteristics of the recovery response in both tolerant and susceptible genotypes, to evaluate the effect of these transitory stresses on plant productivity and investigate whether a transitory stress can provide a sort of “memory” for subsequent stressful events of the same kind.

### Conflict of interest statement

The authors declare that the research was conducted in the absence of any commercial or financial relationships that could be construed as a potential conflict of interest.
