# Supplementary material for: Time course of biochemical, physiological, and molecular responses to field-mimicked conditions of drought, salinity, and recovery in two maize lines
Source: Front Plant Sci. 2015 May 12;6:314. doi: 10.3389/fpls.2015.00314 (PMC4429227; doi:10.3389/fpls.2015.00314)
Supplement: Supplementary file 1 [file Table1.PDF]

Supplemental table 1. Primers used for qRT-PCR expression analysis performed in the maize leaf tissue.

Gene	Primer	Primer sequence (5'->3')	(Genbank Acc. No. or MaizeGDB Acc. No.)*	Reference**
ZmGAPC2	sense	AATGGCAAGCTCACTGGC	GRMZM2G180625	
	antisense	CTGTCACCGGTGAAGTCG		
ZmLEA3	sense	GTCCGTGACCCTGTTTGC	NM_001153473	[1]
	antisense	CCGCCCGACTCGTTTA		
ZmPMP3-4	sense	TTCTGGATCGACCTCTTGCT	EU954642.1	
	antisense	TCCTCCTCTTCGCACAACTT		
ZmHSP70	sense	GATCCCCTCAAGCTCCTTCAT	CA404511	[2]
	antisense	AGATCGAAGATGCCGTTGACA		
ZmCAT1	sense	CCTGTGGTACAAACCCTGCT	NM_001111945.1	
	antisense	ATCCTTGCTGCATCTGTCCG		
ZmPP2C	sense	CTGATGATATCAGTGTCGTGATCGTGCAG	EF195257.1	
	antisense	CGCCAGCGAAGTAACATATCATGTCTACC		
putative B2/DP1 HVA22	sense	ATCCTCACTCACCTCCACTCCCTAGC	GRMZM2G154735	
	antisense	GAGCTCGTACCAGATGGGGATCCAGTAT		
putative calcium-binding EF-hand	sense	TGTCCGCTTGGAGTTCAGTCACTACG	GRMZM5G827398	
	antisense	GAGCTCAGGTTACCATCGCAGTTAGC		
putative hydroxymethylglutaryl-coenzymeA reductase	sense	AGACAAACGTACAGGCTCTCG	CO440726	[2]
	antisense	GCTGCCACAATGTTACTTGC		
ZmSUS	sense	CCCTTCAATGCCTCCTTTCCTC	X02382	[3]
	antisense	TCAACATCATCGTCGTGCCC		
ZmIVR1	sense	GCTGCCTTCCTTATCCTTCTTGTG	U16123.1	[4]
	antisense	CCTGCTCCCTGCTCCTCTTATC		
ZmGLN1	sense	GGCGGGTTTGAAGAGATCAA	NM_001254779.1	[2]
	antisense	CCAGTCAGTCTTCTTTCATTTCCTT		
putative Rab GTPase	sense	ACTAGTGCGTATTACCGAGGCGCTGT	GRMZM2G018619	
	antisense	CGGTAGATCTGAGCTAGGACTTCTGC		
Zmβ-EXP7	sense	CAACCTTGTCCTCCACAGTAG	AF332180	[5]
	antisense	GTGAGGTCGGAGGCGTTAAA		
ZmNHX4 and ZmNHX5	sense	AATCTCTCTCGGCGCAATAG	NM_001112473.1 and NM_001111753.1	
	antisense	CACAGAATCCGTTGCAGAAA		
ZmRMR6	sense	GAGGGTTTGAATCCATTGGAATGTC	NM_001195895.1	[6]
	antisense	GGAGTCCTCTAAACCATTGACCG		
ZmHDA108	sense	AGACTACTACTACGGGCAAG	GRMZM2G136067	
	antisense	CACGCCTGTGGAACTTGAGGAGCTCG		
putative Really Interesting New Gene Zn-finger	sense	GCTCGGCCTCCTCAAGGTTATGCTATAC	GRMZM2G148908	
	antisense	GTTCTCCCTAGTCAAGGTATCCGTGTCC		
putative RNA-binding KH domain-containing protein	sense	GAGTTGAAGCTACTACAGGTTGCCGTGT	AC218972.3_FG007	
	antisense	GGTTTCAGCAATCCTCCAGTATCTC		

*Gene bank numbers according to http://www.ncbi.nlm.nih.gov/. Maize GDB numbers according to http://www.maizegdb.org.

**Primers without reference were designed using Primer BLAST (http://www.ncbi.nlm.nih.gov/tools/primer-blast/). For primer sequences selected from published papers the reference work is reported: [1] Liu Y et al. 2013. [2] under MTA contract with Biogemma, 8 rue des frères Lumière, 63100 Clermont-Ferrand, France. [3] Wang et al. 2003. [4] Kakumanu et al. 2012. [5] Geilfus et al. 2010. [6] primers provided by Dr. V.Rossi (Consiglio per la Ricerca e la Sperimentazione in Agricoltura, Unità di Ricerca per la Maiscoltura, Via Stezzano 24, I-24126 Bergamo, Italy)
