# Supplementary material for: Time course of biochemical, physiological, and molecular responses to field-mimicked conditions of drought, salinity, and recovery in two maize lines
Source: Front Plant Sci. 2015 May 12;6:314. doi: 10.3389/fpls.2015.00314 (PMC4429227; doi:10.3389/fpls.2015.00314)
Supplement: Supplementary file 2 [file Table2.PDF]

**Supplemental table 2.** Relative quantification of gene expression performed with qRT-PCR in the maize leaf tissue.

| | | B73 inbred line | | | | | | | | P26 hybrid | | | | | | | |
|---|---|---|---|---|---|---|---|---|---|---|---|---|---|---|---|---|---|
| | | T10 | | | | T14 | | | | T10 | | | | T14 | | | |
| | | C | WS | SS | WS+SS | C | WS | SS | WS+SS | C | WS | SS | WS+SS | C | WS | SS | WS+SS |
| stress response | *LEA3* | 1 (±0.27) | 1.93 (±0.37) | 83.12 (±14.79) | 25.99 (±4.1) | 3.01 (±0.5) | 1.58 (±0.27) | 7.77 (±1.28) | 1.72 (±0.28) | 1 (±0.07) | 0.85 (±0.08) | 1.98 (±0.46) | 1.62 (±0.15) | 1 (±0.2) | 2.09 (±0.3) | 192.16 (±18.2) | 126.1 (±9.18) |
| | *PMP3-4* | 1 (±0.21) | 1.5 (±0.2) | 871.9 (±116) | 160.48 (±23.15) | 1.74 (±0.35) | 12.31 (±1.6) | 653.37 (±89.15) | 255.53 (±34.21) | 1 (±0.17) | 0.86 (±0.13) | 6.16 (±0.69) | 3.63 (±0.64) | 1.63 (±0.2) | 2.58 (±0.49) | 773.81 (±82.08) | 857.69 (±133) |
| | *HSP70* | 1 (±0.12) | 2.58 (±0.28) | 5.07 (±0.55) | 4.23 (±0.42) | 3.08 (±0.34) | 7.91 (±0.82) | 11.99 (±1.48) | 6.44 (±0.85) | 1 (±0.1) | 2 (±0.2) | 1.9 (±0.17) | 3.08 (±0.24) | 2.05 (±0.21) | 4.22 (±0.42) | 16.01 (±1.57) | 8.12 (±0.74) |
| | *CAT1* | 1 (±0.09) | 0.98 (±0.08) | 3.01 (±0.19) | 1.49 (±0.1) | 1.05 (±0.08) | 1.4 (±0.1) | 2.63 (±0.16) | 1.8 (±0.24) | 1 (±0.06) | 1 (±0.08) | 1.46 (±0.08) | 1.38 (±0.2) | 1.55 (±0.1) | 0.93 (±0.08) | 1.99 (±0.28) | 1.67 (±0.18) |
| ABA response | *PP2C* | 1 (±0.11) | 2.55 (±0.35) | 2.96 (±0.33) | 1.39 (±0.12) | 1.07 (±0.1) | 1.35 (±0.12) | 2.54 (±0.29) | 2.98 (±0.33) | 1 (±0.1) | 1.88 (±0.17) | 0.93 (±0.07) | 4.17 (±0.35) | 2 (±0.2) | 0.83 (±0.08) | 1.69 (±0.12) | 1.47 (±0.15) |
| | *HVA22* | 1 (±0.11) | 9.41 (±0.85) | 4.04 (±0.35) | 10.16 (±0.92) | 3.04 (±0.24) | 8.01 (±0.63) | 37.83 (±2.44) | 15.25 (±0.98) | 1 (±0.44) | 12.74 (±3.18) | 9.43 (±2.43) | 35.24 (±8.35) | 3.75 (±0.95) | 5.74 (±1.55) | 58.46 (±14.19) | 43.4 (±10.61) |
| signalling mechanisms | *EF-Hand* | 1 (±0.27) | 1 (±0.17) | 1.59 (±0.27) | 1.38 (±0.28) | 0.6 (±0.11) | 0.87 (±0.15) | 0.9 (±0.14) | 0.88 (±0.18) | 1 (±0.13) | 1.77 (±0.18) | 1.73 (±0.17) | 1.74 (±0.29) | 0.95 (±0.11) | 1.7 (±0.21) | 2.18 (±0.31) | 1.65 (±0.17) |
| isoprenoids metabolism | *CoAred* | 1 (±0.11) | 1.48 (±0.21) | 4.63 (±0.56) | 2.45 (±0.3) | 1.57 (±0.23) | 1.94 (±0.23) | 3.65 (±0.46) | 2.22 (±0.3) | 1 (±0.15) | 1.13 (±0.13) | 1 (±0.1) | 1.54 (±0.15) | 1.48 (±0.22) | 1.45 (±0.21) | 2.22 (±0.22) | 1.76 (±0.23) |
| carbohydrates metabolism | *SUS* | 1 (±0.09) | 1.31 (±0.09) | 3.39 (±0.24) | 1.26 (±0.12) | 0.81 (±0.08) | 1.03 (±0.07) | 2.21 (±0.15) | 1.17 (±0.07) | 1 (±0.03) | 1.27 (±0.06) | 0.79 (±0.04) | 1.36 (±0.05) | 1.01 (±0.06) | 0.95 (±0.06) | 3.44 (±0.24) | 1.8 (±0.1) |
| | *IVR1* | 1 (±0.17) | 1.34 (±0.24) | 2.09 (±0.32) | 2.15 (±0.32) | 1.96 (±0.25) | 2.96 (±0.47) | 8.01 (±0.96) | 5.16 (±0.65) | 1 (±0.07) | 2.14 (±0.23) | 2.1 (±0.17) | 3.52 (±0.33) | 2.76 (±0.24) | 3.52 (±0.3) | 4.61 (±0.33) | 2.35 (±0.28) |
| nitrogen metabolism | *GLN1* | 1 (±0.08) | 0.57 (±0.04) | 0.64 (±0.07) | 0.76 (±0.07) | 0.44 (±0.02) | 0.42 (±0.05) | 0.51 (±0.03) | 0.41 (±0.03) | 1 (±0.04) | 0.89 (±0.03) | 0.86 (±0.03) | 0.67 (±0.04) | 0.76 (±0.04) | 0.58 (±0.03) | 0.39 (±0.02) | 0.21 (±0.02) |
| membrane trafficking | *Rab GTPase* | 1 (±0.15) | 1.28 (±0.16) | 1.22 (±0.16) | 1.05 (±0.12) | 1.33 (±0.18) | 1.25 (±0.12) | 0.98 (±0.11) | 0.94 (±0.14) | 1 (±0.05) | 0.97 (±0.05) | 0.75 (±0.04) | 0.76 (±0.05) | 0.97 (±0.03) | 1.02 (±0.06) | 1.21 (±0.1) | 0.65 (±0.06) |
| cell wall-loosening mechanism | *β-EXP7* | 1 (±0.21) | 1.91 (±0.37) | 1.32 (±0.21) | 0.79 (±0.11) | 11.36 (±1.59) | 2.74 (±0.4) | 1.98 (±0.44) | 2.1 (±0.52) | 1 (±0.37) | 2.85 (±0.74) | 1 (±0.27) | 1.44 (±0.47) | 1.86 (±0.54) | 2.79 (±0.6) | 1.76 (±0.4) | 0.72 (±0.2) |
| ion transport | *NHX4-5* | 1 (±0.12) | 1.59 (±0.34) | 2.39 (±0.38) | 1.77 (±0.19) | 1.74 (±0.19) | 2.62 (±0.31) | 2.97 (±0.29) | 2.12 (±0.34) | 1 (±0.16) | 0.92 (±0.15) | 0.84 (±0.1) | 0.98 (±0.1) | 1.33 (±0.13) | 0.98 (±0.09) | 1.68 (±0.17) | 2.05 (±0.28) |
| regulation of gene expression | *RMR6* | 1 (±0.2) | 1.54 (±0.21) | 1.25 (±0.24) | 1.13 (±0.21) | 1.2 (±0.17) | 1.29 (±0.2) | 1.62 (±0.27) | 1.22 (±0.17) | 1 (±0.09) | 1.31 (±0.13) | 1.1 (±0.07) | 1.81 (±0.19) | 1.62 (±0.16) | 1.47 (±0.12) | 1.71 (±0.26) | 0.92 (±0.13) |
| | *HDA108* | 1 (±0.11) | 0.91 (±0.13) | 1.03 (±0.09) | 0.88 (±0.07) | 0.72 (±0.08) | 0.75 (±0.05) | 0.91 (±0.06) | 0.69 (±0.07) | 1 (±0.16) | 1.02 (±0.12) | 1.01 (±0.15) | 0.66 (±0.12) | 1.06 (±0.11) | 1.11 (±0.14) | 1.27 (±0.19) | 0.55 (±0.13) |
| protein-protein interaction | *RING* | 1 (±0.18) | 0.85 (±0.11) | 1.09 (±0.17) | 0.67 (±0.1) | 1.26 (±0.19) | 0.88 (±0.19) | 1.93 (±1.23) | 1.27 (±0.15) | 1 (±0.03) | 0.97 (±0.09) | 1.29 (±0.17) | 1.16 (±0.08) | 2.16 (±0.19) | 1.25 (±0.04) | 1.57 (±0.26) | 1.04 (±0.13) |
| RNA-binding | *RNA-binding KH* | 1 (±0.16) | 0.88 (±0.12) | 1.11 (±0.13) | 0.93 (±0.14) | 0.96 (±0.14) | 0.77 (±0.09) | 0.96 (±0.17) | 0.78 (±0.08) | 1 (±0.15) | 0.9 (±0.13) | 0.78 (±0.13) | 0.72 (±0.15) | 1.16 (±0.22) | 0.72 (±0.12) | 0.99 (±0.13) | 0.6 (±0.06) |

Relative quantification of gene expression in maize leaves of B73 inbred line and hybrid P26 at two time points, after 10 days of stress (T10) and 4 days of recovery (T14) following the application of drought (WS), salinity (SS) and drought+salinity (WS+SS). The maize *GAPC2* gene was selected as internal control. Each experiment was run in triplicate. Data from qRT-PCR experiments were analyzed according to the Pfaffl method and gene expression was calculated as the fold change (FC) relative to the expression level of the control non-stressed sample (C) of the same genotype at T10. Numbers are the FC values (±SE).
